# Safety and effectiveness of secukinumab in Japanese patients with generalized pustular psoriasis: A post‐marketing surveillance

**DOI:** 10.1111/1346-8138.17648

**Published:** 2025-04-03

**Authors:** Ayako Fujishige, Noriko Seko

**Affiliations:** ^1^ Analytics and CDM Japan Novartis Pharma K.K Tokyo Japan; ^2^ Biostatistics Novartis Pharma K.K Tokyo Japan

**Keywords:** generalized pustular psoriasis, IL‐17, Japanese, safety, secukinumab

## Abstract

Secukinumab is one of the human monoclonal antibodies recommended in the Japanese guidelines for patients with psoriasis, but few case reports and clinical studies on secukinumab for pustular psoriasis are available because of the rarity of the disease. This was an open‐label, multicenter, uncontrolled, single‐arm, prospective observational surveillance conducted in a clinical practice setting to evaluate the safety and effectiveness of secukinumab in Japanese patients with generalized pustular psoriasis (GPP). Patients were monitored for 1 year after starting secukinumab and followed up for an additional 2 years. Of 99 patients from 71 sites, 95 were included in safety and 82 in effectiveness analysis. The mean (standard deviation) observation period was 346.2 (64.87) days, and 91.58% of patients were observed over 52 weeks. Adverse events, serious adverse events, and adverse reactions were reported in 51.58%, 12.63%, and 35.79% of patients, respectively. Safety evaluations showed no significant difference in the incidence of events based on the history of biologics The proportion of patients with either “complete response” or “partial response” was ~90% from week 2 and remained stable until week 52. The proportion of patients with “remission (no symptom)” in the Japanese Dermatological Association total score increased from week 4 (22.22%) to week 52 (47.83%). The mean Psoriasis Area and Severity Index score decreased from week 1 (17.26) to week 16 (1.18), with the mean percentage change decreasing from −28.07% to −90.18%. The mean Dermatology Life Quality Index (DLQI) total score decreased from 8.7 at the start of secukinumab treatment to 1.9 at week 52. At week 52, the proportion of patients with DLQI total score of 0/1 was 57.14%. No new safety signals for secukinumab in long‐term treatment were observed from this surveillance, and no additional measures needed to be taken. Moreover, secukinumab showed sustained effectiveness in patients with GPP in Japan.

## 1 INTRODUCTION

Psoriasis is a chronic inflammatory skin disease involving multiple organ systems. The prevalence of psoriasis varies from 0.14% in East Asia to 1.99% in Australasia,^1^ while the prevalence in Japan is reported to be 0.34%.^2^ Psoriasis comprises several clinical phenotypes, such as plaque psoriasis, flexural psoriasis, guttate psoriasis, erythrodermic psoriasis, and pustular psoriasis.^3^

Pustular psoriasis is characterized by the occurrence and repeated recurrence of acute or chronic aseptic pustules on skin lesions and can be developed concurrently with psoriasis of other phenotypes.^4^ Pustular psoriasis can be classified into three types: (1) localized pustular psoriasis, called palmoplantar pustular psoriasis (PPP); (2) acrodermatitis continua of Hallopeau, which involves acral areas of the fingers, toes, and nail beds; and (3) generalized pustular psoriasis (GPP), a disseminated, severe, and potentially life‐threatening form of psoriasis.^5^, ^6^ While other phenotypes such as psoriasis vulgaris or plaque psoriasis represent approximately 90% of psoriasis cases,^7^ pustular psoriasis is a rare clinical phenotype. A Japanese epidemiological study reported the prevalence of plaque psoriasis as 97.4%^2^ and the prevalence of pustular psoriasis as 1.1%–7.5%.^2^, ^8^ In 2020, approximately 1900 patients in Japan had GPP.^9^ Although the mechanism of GPP is not fully understood, interleukin (IL)‐17 is thought to contribute to GPP pathogenesis.^10^ IL‐17A is an inflammatory cytokine produced by various cells, including Th17, γδT cells, monocytes, and neutrophils. IL‐17 stimulates the abnormal differentiation of keratinocytes and stimulates the production of pro‐inflammatory cytokines. An observational study revealed that serum IL‐17 levels are significantly higher in patients with GPP and PPP compared to healthy controls, indicating that IL‐17 is involved in the pathogenesis of GPP.^11^ Moreover, a recent study highlighted an upregulation of multiple genes in the IL‐17 signaling pathway and an increased expression of IL‐17A in the epidermis and dermis of GPP skin, further emphasizing the role of IL‐17A in GPP.^12^ The study also found that neutrophil‐derived IL‐17A attracts more neutrophils by upregulating chemokines from keratinocytes via the IL‐17 signaling pathway in GPP skin, leading to an inflammatory crosstalk and neutrophil influx, therefore the inhibition of IL‐17A can be effective in GPP treatment.^13^ Several IL‐17A inhibitors, including secukinumab, are reported to be effective for pustular psoriasis and are listed in the Japanese guidelines for biologics use for psoriasis.^14^

Secukinumab is the first human immunoglobulin (Ig) G1/κ monoclonal antibody targeting human IL‐17A. It was initially approved in December 2014 for treating patients with psoriatic vulgaris and psoriatic arthritis who had not responded adequately to conventional therapies.^15^ Later, in December 2015, the indication for its use was expanded to include pustular psoriasis in patients who did not respond adequately to conventional therapies. However, because of the rarity of pustular psoriasis among patients with psoriasis, case reports and clinical studies on secukinumab use in Japanese patients with GPP are limited.^16^, ^17^, ^18^

This was a post‐marketing surveillance study conducted as instructed by the Japanese Health authority to collect post‐marketing data on secukinumab. The dose and administration of secukinumab was in accordance with the approved label in Japan, that is, subcutaneous administration at a dose of 300 mg at weeks 0, 1, 2, 3 and 4, and at 4‐week intervals afterwards, or at a dose of 150 mg with the same regimen, depending on bodyweight. Data were collected on the clinical use of secukinumab in GPP. The objective of this surveillance was to further evaluate the safety and effectiveness of secukinumab in Japanese patients with GPP.

## 2 METHODS

### 2.1 Study design

This open‐label, multicenter, uncontrolled, single‐arm, prospective, observational surveillance study was conducted in accordance with Good Post‐marketing Study Practice (GPSP) ordinance and the surveillance protocol to evaluate the safety and effectiveness of secukinumab in patients with GPP. Each patient was observed for 52 weeks from the start of secukinumab treatment (observation period), then followed up for 2 years after the end of the observation period (follow‐up period, 156 weeks from the start of secukinumab treatment) for the exclusive collection of specific adverse events (AEs) designated by the health authorities. The surveillance started in January 2016 and ended on November 24, 2021.

### 2.2 Study population

**FIGURE 1:**
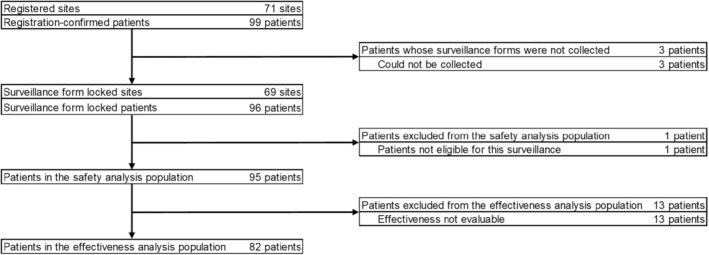
Patient disposition.

Patients with GPP who were inadequately responding to conventional therapies and receiving secukinumab for the first time since its approval for this indication were included in the surveillance. The severity of GPP was assessed according to the Japanese Dermatological Association's severity assessment criteria for GPP. Written consent for participating in the surveillance was obtained before including the patient in the surveillance. Patients with eruption covering 10% or more of the body surface area (BSA) and who were not adequately responding to conventional systemic therapies (excluding biologics), including ultraviolet therapies, as well as those with refractory eruption, joint symptoms, or pustules were included in the surveillance. Patients previously treated with products containing secukinumab as an active ingredient (either as an investigational drug or in post‐marketing surveillance) were excluded from this surveillance. After meeting the eligibility criteria, patients were required to complete a registration form to be included as confirmed patients in the registration process. Subsequently, patients were categorized and included in the safety analysis population and effectiveness analysis population for further evaluation, as shown in the flowchart presented in Figure 1.

### 2.3 Assessments

Safety assessments included the incidences of AEs, serious AEs (SAEs), adverse reactions (AEs for which a causal relationship with secukinumab could not be ruled out by the investigator), serious adverse reactions, events that correspond to the priority investigation items (serious infections, tuberculosis, neutropenia, fungal infections, hypersensitivity reactions, malignant tumors, inflammatory bowel disease [IBD], and cardiovascular/cerebrovascular events), laboratory test results, and other safety evaluations (implementation status of hepatitis tests and tuberculosis test). Events defined as identified risks or potential risks in the risk management plan of secukinumab were considered as priority investigation items in the surveillance. Effectiveness assessments included global impression of change (complete response, partial response, no response, progression, and not evaluable), pustular psoriasis severity total score (Japanese Dermatological Association [JDA] total score) and severity category (remission [no symptom], mild, moderate, and severe), Psoriasis Area and Severity Index (PASI) score and PASI50/75/90/100 responses, BSA (%), Dermatology Life Quality Index (DLQI) total score, and proportion of DLQI total score with 0 or 1.^19^, ^20^

### 2.4 Statistical analysis

The safety analysis period was the observation period (52 weeks from the start date of treatment with secukinumab). Safety analysis population and effectiveness analysis population were defined and used for safety or effectiveness variables, respectively. Descriptive statistics were used to summarize the safety and effectiveness variables. No formal statistical analysis was performed. The incidences of adverse reactions including odds ratio (OR) by patient characteristics (safety analysis population) were calculated. Similarly, the response rates of PASI50/75/90/100 and the proportion of patients with DLQI total score of 0 or 1 were calculated at each evaluation time point along with their respective 95% confidence intervals (CIs).

## 3 RESULTS

### 3.1 Patient disposition and baseline characteristics

The disposition of patients in the surveillance is shown in Figure 1. This surveillance included 99 registration‐confirmed patients from 71 sites. Three of them whose surveillance form could not be collected were excluded, and the surveillance form for the remaining 96 patients was locked. The safety analysis population consisted of 95 patients after excluding one patient who was not eligible for the surveillance. The effectiveness analysis population consisted of 82 patients, excluding 13 patients from the safety analysis population. The details of surveillance study discontinuation are listed in Supporting Information Table S1. A total of nine patients (9.47%) discontinued the study and the most common reason was “failure to return before completion (including hospital transfer)” (*n* = 5, 5.26%).

Patient demographics and disease characteristics of the safety analysis population (*n* = 95) are presented in Table 1. Females were the majority in the population (*n* = 53, 55.79%). The mean (standard deviation [SD]) age of patients was 56.9 years (16.02 years), and the majority of patients were aged ≥15 years to <65 years (*n* = 61, 64.21%). Patients with a duration of psoriasis (time elapsed from the initial onset of psoriasis) of ≥10 to <20 years were the majority (*n* = 22, 23.16%), followed by ≥30 years (*n* = 19, 20.00%). For GPP, a duration of less than 1 year was the most common (*n* = 30, 31.58%), followed by ≥1 to <5 years (*n* = 15, 15.79%). The mean (SD) body weight was 61.31 kg (13.874 kg), and the number of patients weighing ≤60 kg (*n* = 45, 47.37%) was slightly higher than that of patients weighing >60 kg (*n* = 40, 42.11%). Mean (SD) body mass index (BMI) was 24.00 kg/m^2^ (4.788 kg/m^2^) and patients with a BMI of ≥18.5 to <25.0 kg/m^2^ were the majority (*n* = 42, 44.21%), followed by those with a BMI of ≥25.0 to <35.0 kg/m^2^ (*n* = 25, 26.32%). The majority of patients (*n* = 64, 67.37%) presented with a comorbidity, with cardiovascular/cerebrovascular events being the most common (*n* = 19, 20.00%). Most patients (*n* = 93, 97.89%) had a history of pharmacotherapy, with 37 patients (38.95%) using biologics and 85 patients (89.47%) using non‐biologics for psoriasis treatment. Phototherapy for GPP prior to secukinumab treatment was received by 19 patients (20.00%) and narrow band UVB was the most common therapy (*n* = 15, 15.79%). The majority of patients had a baseline PASI score of ≤20 (*n* = 44, 46.32%) prior to secukinumab treatment, whereas 17 patients (17.89%) had a PASI score of >20.

**TABLE 1:** Demographics and disease characteristics (safety analysis population).

Characteristics	Category	*N* = 95
Sex, *n* (%)	Male	42 (44.21)
	Female	53 (55.79)
Age, *n* (%)	<15 years	0 (0.00)
	≥15 to <65 years	61 (64.21)
	≥65 years	34 (35.79)
Age (years)	Mean (SD)	56.9 (16.02)
	Median (range)	59.0 (16–85)
Visit category, *n* (%)	Outpatient	78 (82.11)
	Inpatient	17 (17.89)
Psoriasis duration, *n* (%)	<1 year	9 (9.47)
	≥1 to <5 years	13 (13.68)
	≥5 to <10 years	12 (12.63)
	≥10 to <20 years	22 (23.16)
	≥20 to <30 years	15 (15.79)
	≥30 years	19 (20.00)
	Unknown/not recorded	5 (5.26)
GPP duration, *n* (%)	<1 year	30 (31.58)
	≥1 to <5 years	15 (15.79)
	≥5 to <10 years	11 (11.58)
	≥10 to <20 years	13 (13.68)
	≥20 to <30 years	13 (13.68)
	≥30 years	8 (8.42)
	Unknown/not recorded	5 (5.26)
Weight, *n* (%)	≤60.0 kg	45 (47.37)
	>60.0 kg	40 (42.11)
	Unknown/not recorded	10 (10.53)
Weight (kg), *n* = 85	Mean (SD)	61.31 (13.87)
	Median (range)	59.00 (39.0–97.0)
BMI (kg/m^2^), *n* = 78	Mean (SD)	24.00 (4.79)
	Median (range)	23.18 (16.5–39.1)
Drinking habit, *n* (%)	Habitual drinker (drinking on a daily basis)	6 (6.32)
	Occasional drinker	18 (18.95)
	Nondrinker	41 (43.16)
	Unknown/not recorded	30 (31.58)
Smoking history, *n* (%)	Current smoker	12 (12.63)
	Ex‐smoker (no longer a smoker)	9 (9.47)
	Nonsmoker	44 (46.32)
	Unknown/not recorded	30 (31.58)
Medical history, *n* (%)	Yes	44 (46.32)
Concomitant medical condition, *n* (%)	Yes	64 (67.37)
Serious infections	Yes	0 (0.00)
Fungal infections	Yes	5 (5.26)
Tuberculosis	Yes	0 (0.00)
Neutropenia	Yes	1 (1.05)
Hypersensitivity reactions	Yes	10 (10.53)
Malignant tumor	Yes	3 (3.16)
Inflammatory bowel disease	Yes	1 (1.05)
CV/cerebrovascular events	Yes	19 (20.00)
Previous drugs, *n* (%)	Yes	93 (97.89)
Biological products (for psoriasis treatment and others)	Yes	38 (40.00)
Biological products (for psoriasis treatment)	Yes	37 (38.95)
Phototherapy, *n* (%)^a^	Yes	19 (20.00)
Oral PUVA	Yes	0 (0.00)
External PUVA	Yes	2 (2.11)
PUVA bath	Yes	3 (3.16)
UVB	Yes	1 (1.05)
Narrow band UVB	Yes	15 (15.79)
Others	Yes	1 (1.05)
GCAP, *n* (%)^b^	Yes	10 (10.53)
PASI score, *n* (%)	≤20	44 (46.32)
	>20	17 (17.89)
	Unknown/not specified	34 (35.79)

Abbreviations: BMI, body mass index; CV, cardiovascular; GCAP, granulocyte and monocyte adsorption apheresis; GPP, generalized pustular psoriasis; *N*, total number of patients; *n*, number of evaluable patients; PASI, Psoriasis Area and Severity Index; PUVA, psoralen plus ultraviolet A; SD, standard deviation; UVB, ultraviolet B.

^a^ Phototherapy for GPP before secukinumab treatment.

^b^ GCAP for psoriasis before secukinumab treatment.

### 3.2 Secukinumab administration

The observation period and exposure to secukinumab in the safety analysis population (*n* = 95) are shown in Supporting Information Table S2. The mean (SD) observation period was 346.2 days (64.87 days) and most patients (*n* = 87, 91.58%) were observed over 48 weeks. The mean (SD) duration of secukinumab treatment was 278.1 days (120.41 days) and 58 patients (61.05%) received secukinumab for over 48 weeks. The majority of patients (*n* = 90, 94.74%) started with 300 mg as the first dose. The mean (SD) total number of administrations was 13.1 (4.32) and 44 patients (46.32%) experienced at least one self‐administration.

The number of secukinumab administrations up to week 16 is shown in Table 2. Overall, 76 of 89 patients (85.39%) who continued secukinumab treatment for more than 4 weeks had five administrations and 66 of 82 patients (80.49%) who continued secukinumab treatment for more than 16 weeks had eight administrations.

**TABLE 2:** Number of secukinumab administrations (up to week 16; safety analysis population).

Secukinumab treatment duration	Number of administrations	n (%)^a^, *N* = 95
>4 weeks		89 (93.68)
	<5	13 (14.61)
	5	76 (85.39)
	>5	0 (0.00)
>16 weeks		82 (86.32)
	<8	13 (15.85)
	8	66 (80.49)
	>8	1 (1.22)
	Unknown/not recorded	2 (2.44)

Abbreviations: *N*, total number of patients; *n*, number of evaluable patients.

^a^ The safety analysis population (*N*) was used as denominator to calculate the proportions of patients who continued secukinumab treatment for 4 weeks and for 16 weeks. Number of patients continuing secukinumab treatment was used as the denominator to calculate the proportions of the number of administrations.

Secukinumab dose was changed in eight patients (8.42%) and the details of dose changes are shown in Table 3. Dose reduction was implemented in five patients (5.26%), with the occurrence of AE being the most common reason (*n* = 4, 4.21%). Among four patients (4.21%) who had a dose increase, lack of effectiveness was the most common reason (*n* = 2, 2.11%).

**TABLE 3:** Dose changes (safety analysis population).

	*n* (%)*, *N* = 95
Patients with dose change	8 (8.42)
Patients with dose reduction^a^	5 (5.26)
Reason for reduction (*n* ≥ 2)	
Adequate efficacy	2 (2.11)
Occurrence of adverse events	4 (4.21)
Patient with dose increase^b^	4 (4.21)
Reason for increase (*n* ≥ 2)	
Lack of effectiveness	2 (2.11)

Abbreviations: *N*, total number of patients; *n*, number of evaluable patients.

* Patients with multiple dose changes are counted for each reason.

^a^ Dose reduction: from secukinumab 300 mg to 150 mg.

^b^ Dose increase: from secukinumab 150 mg to 300 mg.

The reasons for switching to secukinumab and the number of days from the last date of biologics prior to secukinumab treatment are shown in Supporting Information Tables S3 and S4. Out of 38 patients (40.00%) who received treatment with biologics prior to secukinumab treatment, infliximab was the most common prior biologic (*n* = 35/38) received and used for GPP in the majority of patients (*n* = 34/35, 97.14%). The most common reason for switching from infliximab to secukinumab was lack of effectiveness (*n* = 18/35, 51.43%).

Concomitant therapies used during the observation period and the secukinumab treatment period are shown in Supporting Information Table S5. Topical steroid was the most commonly used non‐biologic psoriatic medication during secukinumab treatment (*n* = 54, 56.84%). Concomitant therapies performed during the observation period included phototherapy for GPP in two patients (2.11%) and granulocyte and monocyte adsorption apheresis for GPP in five patients (5.26%). Concomitant therapies for purposes other than GPP treatment were performed in four patients (4.21%) during the observation period.

### 3.3 Safety

The safety of secukinumab was evaluated in the safety analysis population (*n* = 95).

Incidences of AEs are summarized in Table 4. AEs were reported in 49 patients (51.58%). The common AEs (≥3.00%) included GPP (verbatim: worsening of primary disease) in nine patients (9.47%), followed by folliculitis, oral candidiasis, interstitial lung disease, upper respiratory tract inflammation, rash, and pyrexia in three patients each (3.16%).

**TABLE 4:** Incidence of AEs by SOC, PT (≥2 patients, safety analysis population).

SOC PT	Number of patients with AE (%), *N* = 95
Total	49 (51.58)
Infections and infestations	16 (16.84)
Folliculitis	3 (3.16)
Oral candidiasis	3 (3.16)
Nasopharyngitis	2 (2.11)
Skin candida	2 (2.11)
Neoplasms benign, malignant, and unspecified (including cysts and polyps)	3 (3.16)
Blood and lymphatic system disorders	2 (2.11)
Metabolism and nutrition disorders	2 (2.11)
Nervous system disorders	3 (3.16)
Headache	2 (2.11)
Vascular disorders	2 (2.11)
Hypertension	2 (2.11)
Respiratory, thoracic, and mediastinal disorders	7 (7.37)
Interstitial lung disease	3 (3.16)
Upper respiratory tract infection	3 (3.16)
Gastrointestinal disorders	4 (4.21)
Hepatobiliary disorders	3 (3.16)
Liver disorder	2 (2.11)
Skin and subcutaneous tissue disorders	18 (18.95)
GPP	9 (9.47)
Rash	3 (3.16)
Drug eruption	2 (2.11)
Erythema	2 (2.11)
Pruritus	2 (2.11)
General disorders and administration site conditions	7 (7.37)
Pyrexia	3 (3.16)
Investigations	10 (10.53)
Gamma‐glutamyl transferase increased	2 (2.11)
Neutrophil count decreased	2 (2.11)
White blood cell count decreased	2 (2.11)

*Note*: A patient with multiple events (PT) was counted only once. SOCs are shown in the order of international consensus. PT is shown in the descending order of incidences, followed by the order of PT codes. MedDRA/J version 24.1.

Abbreviations: AE, adverse event; GPP, generalized pustular psoriasis; *N*, total number of patients; PT, preferred term; SOC, system organ class.

AEs were reported in 17 of 36 patients (47.22%) with a history of biologics use, and in 32 of 59 patients (54.24%) without a history of biologic use. The common AEs (≥3.00%) in patients with a history of biologics use were GPP (verbatim: worsening of primary disease, *n* = 3/36, 8.33%) and oral candidiasis, skin candida, liver disorder, and erythema (*n* = 2/36, 5.56% each). The common AEs (≥3.00%) in patients without a history of biologic use were GPP (verbatim: worsening of primary disease, *n* = 6/59, 10.17%), interstitial lung disease, and pyrexia (*n* = 3/59, 5.08% each); and folliculitis, headache, hypertension, upper respiratory tract inflammation, rash, drug eruption, decrease in neutrophil count, and decrease in white blood cell count (*n* = 2/59, 3.39% each).

A total of 16 patients (16.84%) reported AEs that led to discontinuation of secukinumab. These events comprised various conditions, including GPP (verbatim: worsening of primary disease) in 4.21% (four patients) as well as nasopharyngitis, pneumonia staphylococcal, sepsis, hepatocellular carcinoma, interstitial lung disease, liver disorder, drug eruption, erythema, pruritus, rash, drug ineffective, decrease in therapeutic response, decrease in neutrophil count, decrease in white blood cell count, and Krebs von den Lungen‐6 increased in one patient each (1.05%). The outcomes of liver disorder were resolving or resolved, except for one case of death. Discontinuation of secukinumab due to AEs was comparable between patients with and without a history of biologic use (16.67% [*n* = 6/36] and 16.95% [*n* = 10/59], respectively).

The incidence of SAEs is shown in Supporting Information Table S6. SAEs were reported in 12 patients (12.63%) and none of the SAEs were reported in more than two patients. One patient developed hepatocellular carcinoma 149 days after the start of secukinumab treatment (9 days after the last dose) and died after 1234 days after the onset. Secukinumab treatment was discontinued and a causal relationship with secukinumab was ruled out by the investigators. The incidence of SAEs reported in patients with and without a history of biologic use were 11.11% (*n* = 4/36) and 13.56% (*n* = 8/59), respectively. The common SAEs reported in patients with a history of biologic use were oral candidiasis, septic shock, pancreatic carcinoma recurrent, hepatocellular carcinoma, erythema, and pruritus (*n* = 1/36, 2.78% each). The common SAEs reported in patients without a history of biologic use were pneumonia staphylococcal, sepsis, tonsillitis, breast cancer stage II, adrenal insufficiency, colitis ulcerative, intestinal obstruction, and pyrexia (*n* = 1/59, 1.69% each). The incidences of SAEs showed no significant difference between patients with and without a history of biologic use.

The incidences of adverse reactions are summarized in Supporting Information Table S7. Adverse reactions were reported in 34 patients (35.79%). The common adverse reactions (≥3.00%) observed were GPP (verbatim: worsening of primary disease, *n* = 4, 4.21%) and oral candidiasis and rash (*n* = 3, 3.16% each). Among the 34 patients who experienced adverse reactions, the majority (11 patients) reported these reactions within 4 weeks of starting the treatment. This was followed by six patients who reported the reactions during >4 and ≤16 weeks after treatment initiation. No unique adverse reaction occurring at a specific time point was reported.

Incidences of adverse reactions reported in patients with and without a history of biologic use were 27.78% (*n* = 10/36) and 40.68% (*n* = 24/59), respectively. Oral candidiasis (*n* = 2/36, 5.56%) was the most common adverse reaction in patients with a history of biologic use, and the common adverse reactions (≥3.00%) in patients without a history of biologic use were GPP (verbatim events: worsening of the primary disease, *n* = 4/59, 6.78%), as well as hypertension, rash, decrease in neutrophil count, and decrease in white blood cell count (*n* = 2/59, 3.39% each).

Serious adverse reactions were reported in six patients (6.32%), and these were oral candidiasis, pneumonia staphylococcal, sepsis, septic shock, breast cancer stage II, colitis ulcerative, erythema, and pruritus (*n* = 1, 1.05% each).

As 95% CIs of the OR include the value 1 for all factors, no statistically significant difference between the characteristics was observed.

The incidences of priority investigation items (AEs and adverse reactions) during the observation period in the safety analysis population are summarized in Table 5. Each priority investigation item is described in the following section and no AEs of tuberculosis occurred.

**TABLE 5:** Incidence of priority investigation items by PT (AEs and adverse reactions) (safety analysis population).

	*N* = 95	
Priority investigation items	AEs	Adverse reactions
PT	*n* (%)	*n* (%)
Total	24 (25.26)	18 (18.95)
Serious infections	4 (4.21)	3 (3.16)
Oral candidiasis	1 (1.05)	1 (1.05)
Pneumonia staphylococcal	1 (1.05)	1 (1.05)
Sepsis	1 (1.05)	1 (1.05)
Septic shock	1 (1.05)	1 (1.05)
Tonsilitis	1 (1.05)	0 (0.00)
Fungal infections	8 (8.42)	6 (6.32)
Oral candidiasis	3 (3.16)	3 (3.16)
Skin candida	2 (2.11)	1 (1.05)
Malassezia infection	1 (1.05)	0 (0.00)
*Tinea* infection	1 (1.05)	1 (1.05)
*Candida* infection	1 (1.05)	1 (1.05)
Tuberculosis	0 (0.00)	0 (0.00)
Neutrophil count decreased	2 (2.11)	2 (2.11)
Neutrophil count decreased	2 (2.11)	2 (2.11)
White blood cell count decreased	2 (2.11)	2 (2.11)
Hypersensitivity reactions	6 (6.32)	5 (5.26)
Rash	3 (3.16)	3 (3.16)
Drug eruption	2 (2.11)	1 (1.05)
Allergic conjunctivitis	1 (1.05)	0 (0.00)
Urticaria	1 (1.05)	1 (1.05)
Malignant tumors	3 (3.16)	1 (1.05)
Breast cancer stage II	1 (1.05)	1 (1.05)
Recurrent pancreatic carcinoma	1 (1.05)	0 (0.00)
Hepatocellular carcinoma	1 (1.05)	0 (0.00)
Inflammatory bowel disease	1 (1.05)	1 (1.05)
Colitis ulcerative	1 (1.05)	1 (1.05)
Cardiovascular/cerebrovascular events	2 (2.11)	2 (2.11)
Hypertension	2 (2.11)	2 (2.11)

*Note*: A patient with multiple events (PT) was counted only once. Priority investigation items (in the order of appearance in surveillance form), PT is shown in the descending order of incidences in the adverse event column, followed by the order of PT codes. MedDRA/J version 24.1.

Abbreviations: AE, adverse event; *N*, total number of patients; *n*, number of patients; PT, preferred term.

### 3.4 Serious infection

Serious infections were reported in 4.21% (*n* = 4) and 3.16% (*n* = 3) of patients as AEs and adverse reactions, respectively. AEs reported as serious infections include oral candidiasis, pneumonia staphylococcal, sepsis, septic shock, and tonsillitis (*n* = 1, 1.05% each), and adverse reactions reported as serious infections included oral candidiasis, pneumonia staphylococcal, sepsis, and septic shock (*n* = 1, 1.05% each).

The median (range) number of days from the start of secukinumab treatment to the occurrence of an adverse reaction (first occurrence) was 54.0 days (11–170 days). In patients with adverse reactions, the outcomes were resolved or resolving, and the median (range) number of days to resolved or resolving was 33.0 days (28–129 days). A serious infection that occurred during the follow‐up period was aspiration pneumonia (one patient). The event occurred 1006 days after the start of secukinumab treatment (986 days after prior treatment) and was resolved 18 days after the occurrence by hospitalization/extended hospitalization. Investigators assessed this event to be serious; however, a causal relationship between secukinumab was ruled out.

The incidences of serious infections reported as AEs in patients with and without a history of biologic use were 2.78% (*n* = 1/36) and 5.08% (*n* = 3/59), respectively, and those of adverse reactions in patients with and without a history of biologic use were 2.78% (*n* = 1/36) and 3.39% (*n* = 2/59), respectively.

### 3.5 Neutropenia

The incidences of AEs and adverse drug reactions of decreased neutrophil count were 2.11% each (*n* = 2) and those of decreased neutrophil count and decreased white blood cell count were 2.11% each (*n* = 2).

The median number of days (range) from the start of secukinumab treatment to the occurrence of an adverse reaction (first occurrence) was 30.5 days (29–32 days). The outcomes were resolved or resolving, and median number of days (range) to resolved or resolving was 152.0 days (67–237 days).

No neutropenia as AE or adverse reaction was reported in patients with a history of biologic use. The incidence of neutropenia reported as an adverse reaction in patients without history of biologics use was 3.39%, which includes decrease in neutrophil count and decrease in white blood cell count (*n* = 2/59, 3.39% each). The outcomes were resolved or resolving.

### 3.6 Fungal infections

Fungal infections were reported as AEs and adverse reactions in 8.42% (*n* = 8) and 6.32% (*n* = 6) of patients, respectively. Fungal infections reported as AEs include oral candidiasis (*n* = 3, 3.16%), skin candida (*n* = 2, 2.11%), Malassezia infection, *Tinea* infection, and *Candida* infection (*n* = 1, 1.05% each). Fungal infections reported as adverse reactions include oral candidiasis (*n* = 3, 3.16%), skin candida, *Tinea* infection, and *Candida* infection (*n* = 1, 1.05% each).

The median number of days (range) from the start of secukinumab treatment to the occurrence of adverse reaction (first occurrence) was 141.5 days (92–337 days). The outcomes were resolved or resolving, and the median number of days (range) to resolved or resolving was 85.0 days (15–162 days).

Incidences of fungal infections reported as AEs in patients with and without a history of biologic use were 13.89% (*n* = 5/36) and 5.08% (*n* = 3/59), respectively, and adverse reactions in patients with and without a history of biologic use were 11.11% (*n* = 4/36) and 3.39% (*n* = 2/59), respectively.

### 3.7 Hypersensitivity reactions

Hypersensitivity reactions were reported as AEs and adverse reactions in 6.32% (*n* = 6) and 5.26% (*n* = 5) of patients, respectively. Hypersensitivity reactions reported as AEs include rash (*n* = 3, 3.16%), drug eruption (*n* = 2, 2.11%), and allergic conjunctivitis and urticaria (*n* = 1, 1.05% each). Adverse reactions reported as hypersensitivity reactions include rash (*n* = 3, 3.16%) and drug eruption and urticaria (*n* = 1, 1.05% each).

The median number of days (range) from the start of secukinumab treatment to the occurrence of adverse reaction (first occurrence) was 132.0 days (8–311 days). The outcomes were resolved or resolving, and the median number of days (range) to resolved or resolving was 24.5 days (8–71 days).

Incidences of hypersensitivity reactions reported as AEs in patients with and without a history of biologic use were 2.78% (*n* = 1/36) and 8.47% (*n* = 5/59), respectively, and those of adverse reactions in patients with and without a history of biologic use were 2.78% (*n* = 1/36) and 6.78% (*n* = 4/59), respectively.

### 3.8 Malignant tumors

Malignant tumors were reported as AEs and adverse reactions in 3.16% (*n* = 3) and 1.05% (*n* = 1) of patients, respectively. Malignant tumors reported as AEs include breast cancer stage II, pancreatic carcinoma recurrent, and hepatocellular carcinoma (*n* = 1, 1.05% each). One case of malignant tumor reported as an adverse reaction was breast cancer stage II. The event occurred 21 days after the start of secukinumab treatment (1 day after the last dose) and was assessed to be serious, and after taking measures (observation, change in concomitant medication, hospitalization/extend hospitalization), the outcome was unknown. No secukinumab was administered after the observation and the total number of administrations was four. Small intestine carcinoma (reported as a malignant tumor) developed during the follow‐up period in the same patient who developed breast cancer stage II during the observation period. The event occurred 374 days after the start of secukinumab treatment (354 days after prior treatment), and after taking measures (change in concomitant medication, hospitalization/extend hospitalization, unknown), the outcome was unknown. The event was assessed to be serious and causally related to secukinumab.

Incidences of malignant tumors reported as AEs in patients with and without a history of biologic use were 5.56% (*n* = 2/36) and 1.69% (*n* = 1/59) of patients, respectively, and that of adverse reactions in patients with a history of biologics use was 1.69% (*n* = 1/59).

### 3.9 Inflammatory bowel disease

IBD was reported as an AE and an adverse reaction in 1.05% of patients (*n* = 1). The number of days from the start of secukinumab treatment to the occurrence of adverse reactions (first occurrence) was 54 days and the number of days to resolved was 40 days. No IBD was reported as an AE or adverse reaction in patients with a history of biologic use. IBD was reported as an adverse reaction in 1.69% of patients (one of 59) without a history of biologic use and the outcome was resolved.

### 3.10 Cardiovascular/cerebrovascular events

Cardiovascular/cerebrovascular events were reported as AE and an adverse reaction in 2.11% of patients (*n* = 2). The number of days from the start of secukinumab treatment to the occurrence of adverse reaction (first occurrence) was 1 day and 227 days, and the number of days to resolving was 29 days and 99 days. No cardiovascular/cerebrovascular events were reported as an AE or an adverse reaction in patients with a history of biologic use. These events were reported as an adverse reaction in 3.39% (*n* = 2/59) of patients without a history of biologic use. Two events reported were hypertension and the outcomes were resolving.

### 3.11 Laboratory values

The change in laboratory values including white blood cell count (/μL), neutrophil (%), and C‐reactive protein (CRP) (mg/dL) were evaluated.

The mean (SD) white blood cell count (/μL) started to decrease from week 1 until week 3 (5905.6 [2782.41]), then became stable from week 16 (6747.8 [3321.24]) to week 52 (6386.3 [2496.81]). The mean (SD) neutrophil (%) started to decrease from week 1 until week 4 (55.79 [12.28]), then became stable from week 16 (60.88 [13.50]) to week 52 (59.30 [13.44]). The mean (SD) CRP (mg/dL) kept decreasing from week 1 until week 12 (0.25 [0.31]), then became stable from week 16 (0.48 [1.06]) to week 52 (0.24 [0.31]). The trends in CRP are shown in Supporting Information Figure S1.

### 3.12 Other safety evaluations

In accordance with the important precautions listed in the package insert of secukinumab, the implementation status of hepatitis test and tuberculosis test in the safety analysis population were reviewed and are shown in Supporting Information Table S8. Hepatitis tests were conducted in 46.32% of patients (*n* = 44) and tuberculosis tests were conducted in 71.58% of patients (*n* = 68).

### 3.13 Effectiveness outcomes

The effectiveness of secukinumab was evaluated in the effectiveness analysis population (*n* = 82). Global impressions of change at each time point are shown in Table 6. Among global improvement ratings, either “complete response” or “partial response” was defined as showing response to secukinumab treatment. Response was observed from week 1, and the response rate was stable at around 90% from week 2 to week 52, the rates at weeks 2, 4, 16, and 52 being 92.45% (*n* = 49/53), 93.75% (*n* = 60/64), 94.44% (*n* = 51/54), and 90.00% (*n* = 36/40), respectively. In particular, the proportion of patients with “complete response” increased from week 1 until week 8 and remained stable up to week 52. The proportions at weeks 4, 16, and 52 were 43.75% (*n* = 28/64), 55.56% (*n* = 30/54), and 65.00% (*n* = 26/40) of patients, respectively. The response rate was assessed by patient characteristics. For all characteristics, 95% CIs of the OR included the value 1, implying that none of the characteristics impacted the effectiveness.

**TABLE 6:** Global impression of change (effectiveness analysis population).

Time of evaluation	*N*	Global improvement rating					Response rate^a^	
		Complete response (%)	Partial response (%)	No response (%)	Progression (%)	Not evaluable (%)	*n* (%)	95% CI
Week 1	56	12 (21.43)	35 (62.50)	5 (8.93)	3 (5.36)	1 (1.79)	47 (83.93)	(71.67, 92.38)
Week 2	53	18 (33.96)	31 (58.49)	4 (7.55)	0 (0.00)	0 (0.00)	49 (92.45)	(81.79, 97.91)
Week 3	51	22 (43.14)	28 (54.90)	0 (0.00)	1 (1.96)	0 (0.00)	50 (98.04)	(89.55, 99.95)
Week 4	64	28 (43.75)	32 (50.00)	3 (4.69)	1 (1.56)	0 (0.00)	60 (93.75)	(84.76, 98.27)
Week 8	59	31 (52.54)	23 (38.98)	1 (1.69)	3 (5.08)	1 (1.69)	54 (91.53)	(81.32, 97.19)
Week 12	51	25 (49.02)	24 (47.06)	2 (3.92)	0 (0.00)	0 (0.00)	49 (96.08)	(86.54, 99.52)
Week 16	54	30 (55.56)	21 (38.89)	1 (1.85)	1 (1.85)	1 (1.85)	51 (94.44)	(84.61, 98.84)
Week 24	62	33 (53.23)	24 (38.71)	1 (1.61)	4 (6.45)	0 (0.00)	57 (91.94)	(82.17, 97.33)
Week 36	49	26 (53.06)	18 (36.73)	1 (2.04)	1 (2.04)	3 (6.12)	44 (89.80)	(77.77, 96.60)
Week 52	40	26 (65.00)	10 (25.00)	1 (2.50)	1 (2.50)	2 (5.00)	36 (90.00)	(76.34, 97.21)
Last evaluation^b^	82	40 (48.78)	28 (34.15)	2 (2.44)	5 (6.10)	7 (8.54)	68 (82.93)	(73.02, 90.34)

*Note*: The Clopper–Pearson method was used for the calculation of 95% CI.

Abbreviation: CI, confidence interval; *N*, number of evaluable patients, *n*, number of patients.

^a^ Response: “complete response” or “partial response.”

^b^ Last time point from the day after the start date of secukinumab treatment to week 52.

**FIGURE 2:**
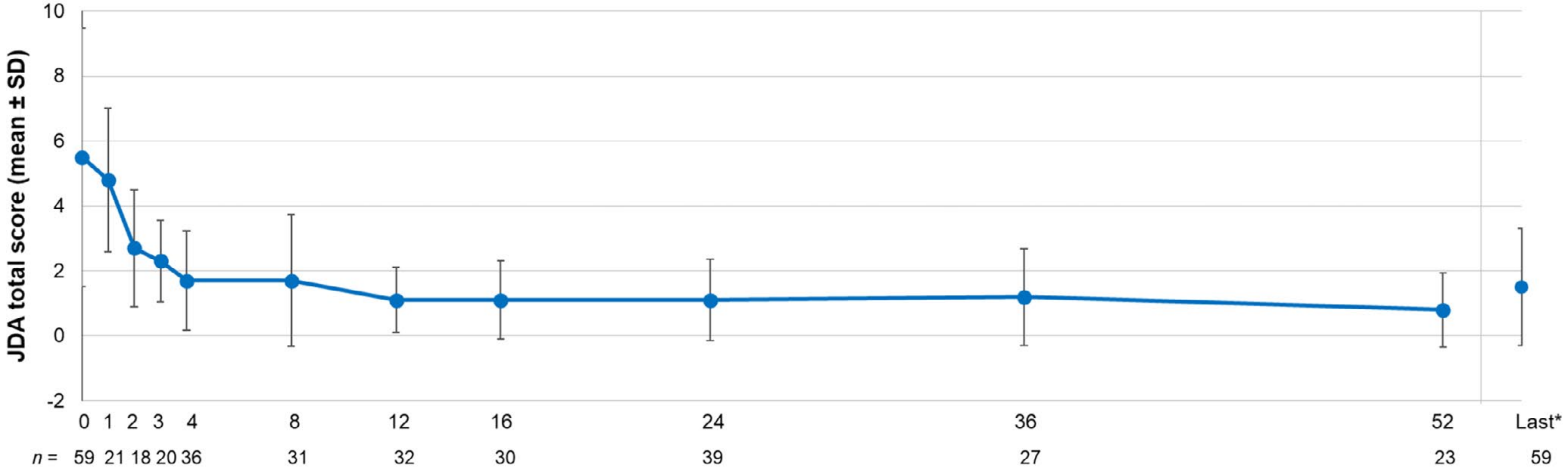
Changes in JDA total score (mean ± SD) in patients from the effectiveness analysis population. *Last time point during 52 weeks of the observational period. JDA, Japanese Dermatological Association; SD, standard deviation.

JDA total scores (category) at each time point and mean (SD) JDA total scores are shown in Table 7 and Figure 2, respectively. Among 59 patients who were evaluable in the effectiveness analysis population, the proportions of patients with JDA total score (category) with “remission (no symptoms),” “mild,” “moderate,” and “severe” at the start of secukinumab treatment were 3.39% (*n* = 2), 59.32% (*n* = 35), 23.73% (*n* = 14), and 13.56% (*n* = 8), respectively. The proportion of patients with “remission (no symptom)” increased from week 4 (*n* = 8/36, 22.22%) to week 16 (*n* = 11/30, 36.67%) and week 52 (*n* = 11/23, 47.83%). The proportions of patients with “mild” at weeks 4, 16, and 52 were 77.78% (*n* = 28/36), 63.33% (*n* = 19/30), and 52.17% (*n* = 12/23), respectively, and no case of “moderate” or “severe” was reported from week 12.

**TABLE 7:** JDA total score (category; effectiveness analysis population).

Time of evaluation		JDA total score (category)^a^			
	*N*	Remission (no symptom) (%)	Mild (%)	Moderate (%)	Severe (%)
At the start of secukinumab treatment	59	2 (3.39)	35 (59.32)	14 (23.73)	8 (13.56)
Week 1	21	0 (0.00)	16 (76.19)	5 (23.81)	0 (0.00)
Week 2	18	2 (11.11)	15 (83.33)	1 (5.56)	0 (0.00)
Week 3	20	2 (10.00)	18 (90.00)	0 (0.00)	0 (0.00)
Week 4	36	8 (22.22)	28 (77.78)	0 (0.00)	0 (0.00)
Week 8	31	10 (32.26)	20 (64.52)	1 (3.23)	0 (0.00)
Week 12	32	10 (31.25)	22 (68.75)	0 (0.00)	0 (0.00)
Week 16	30	11 (36.67)	19 (63.33)	0 (0.00)	0 (0.00)
Week 24	39	16 (41.03)	23 (58.97)	0 (0.00)	0 (0.00)
Week 36	27	10 (37.04)	17 (62.96)	0 (0.00)	0 (0.00)
Week 52	23	11 (47.83)	12 (52.17)	0 (0.00)	0 (0.00)
Last evaluation^b^	59	16 (27.12)	42 (71.19)	1 (1.69)	0 (0.00)

Abbreviations: JDA, Japanese Dermatological Association; *N*, number of evaluable patients.

^a^ Remission (no symptoms) = 0, mild = 1–6, moderate = 7–10, severe = 11–17.

^b^ Last time point from the day after the start of secukinumab treatment to week 52. Patients with data at start of secukinumab treatment and the last evaluation were included.

**FIGURE 3:**
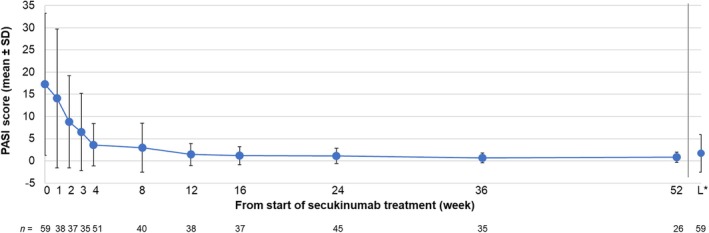
Changes in PASI score (mean ± SD) in patients from the effectiveness analysis population. *Last time point during 52 weeks of the observational period. PASI, Psoriasis Area and Severity Index; SD, standard deviation.

**FIGURE 4:**
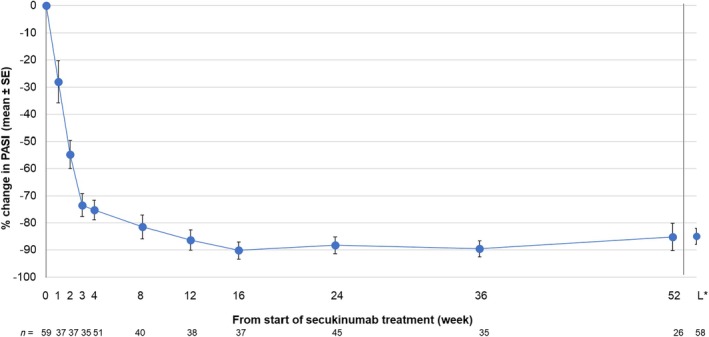
Changes (percentage change) in PASI score (mean ± SE) in patients from the effectiveness analysis population. *Last time point during 52 weeks of the observational period. PASI, Psoriasis Area and Severity Index; SE, standard error.

Changes in mean (SD) PASI score and mean (SD) percentage change in PASI score are shown in Figures 3 and 4, respectively. Mean (SD) PASI score at the start of secukinumab treatment was 17.26 (16.03); the score continuously decreased from week 1, reaching 3.63 (4.78) at week 4 and 1.18 (2.03) at week 16. The mean (SD) PASI score remained stable after week 16 and was 0.83 (1.15) at week 52. The mean percentage change in PASI score showed a continuous decrease until week 16, then became stable. The mean (SD) percentage change in PASI scores at weeks 1, 2, 3, 4, 16, and 52 was −28.07% (7.82), −54.78% (5.22), −73.45% (4.32), −75.23% (3.57), −90.18% (3.11), and − 85.20% (5.08), respectively.

The PASI50/75/90/100 response rates of 59 patients from the effectiveness analysis population whose PASI scores were available are summarized in Table 8. Overall, the PASI response rate started to increase from week 1 and was maintained from weeks 16–52. The proportions of PASI 75/90/100 at week 16 (*n* = 37) and at week 52 (*n* = 26) were 89.19%/75.68%/45.95% (*n* = 33//28/17) and 76.92%/65.38%/46.15% (*n* = 20/17/12), respectively.

**TABLE 8:** PASI 50/75/90/100 response rates (effectiveness analysis population).

Time of evaluation	Response criteria	*N*	Responder	
			*n* (%)	95% CI
Week 16	PASI 50	37	36 (97.30)	(85.84, 99.93)
	PASI 75	37	33 (89.19)	(74.58, 96.97)
	PASI 90	37	28 (75.68)	(58.80, 88.23)
	PASI 100	37	17 (45.95)	(29.49, 63.08)
Week 36	PASI 50	35	34 (97.14)	(85.08, 99.93)
	PASI 75	35	28 (80.00)	(63.06, 91.56)
	PASI 90	35	24 (68.57)	(50.71, 83.15)
	PASI 100	35	17 (48.57)	(31.38, 66.01)
Week 52	PASI 50	26	23 (88.46)	(69.85, 97.55)
	PASI 75	26	20 (76.92)	(56.35, 91.03)
	PASI 90	26	17 (65.38)	(44.33, 82.79)
	PASI 100	26	12 (46.15)	(26.59, 66.63)
Last evaluation^a^	PASI 50	59	51 (86.44)	(75.02, 93.96)
	PASI 75	59	45 (76.27)	(63.41, 86.38)
	PASI 90	59	36 (61.02)	(47.44, 73.45)
	PASI 100	59	24 (40.68)	(28.07, 54.25)

*Note*: The Clopper–Pearson method was used for the calculation of 95% CI.

Abbreviations: CI, confidence interval; *N*, number of evaluable patients, *n*, number of patients; PASI, Psoriasis Area and Severity Index.

^a^ Last time point from the day after the start of secukinumab treatment to week 52. Patients with data at the start of secukinumab treatment and the last evaluation were included.

In 10 patients whose PASI was not assessed, the proportion of BSA (%) was used to evaluate psoriasis and skin symptoms. Mean (SD) BSA at the start of secukinumab treatment and at weeks 4, 16, and 52 was 38.20 (34.77), 18.00 (21.40), 5.80 (4.21), and 1.33 (1.53), respectively.

**FIGURE 5:**
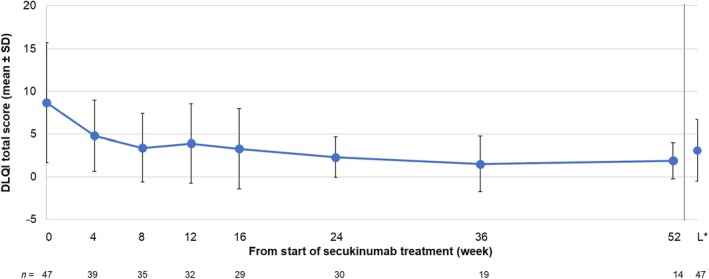
Changes in DLQI total score (mean ± SD) in patients from the effectiveness analysis population. *Last time point during 52 weeks of the observational period. DLQI, Dermatology Life Quality Index; SD, standard deviation.

The DLQI total score in 47 evaluable patients is shown in Figure 5. Mean (SD) DLQI total score was 8.7 (7.03) at the start of secukinumab, and the score started to decrease from week 4, reaching 4.8 (4.14), 3.3 (4.68), and 1.9 (2.13) at weeks 4, 16, and 52, respectively. The proportions of patients with DLQI total score of 0 or 1 at start of secukinumab treatment, week 4, week 16, and week 52 were 17.02%, 30.77%, 51.72%, and 57.14%, respectively (Table 9).

**TABLE 9:** Proportions of DLQI total score = 0 or 1 (effectiveness analysis population).

Time of evaluation	*N*	DLQI total score = 0 or 1	
		*n* (%)	95% CI
At the start of secukinumab treatment	47	8 (17.02)	(7.65, 30.81)
Week 4	39	12 (30.77)	(17.02, 47.57)
Week 8	35	16 (45.71)	(28.83, 63.35)
Week 12	32	12 (37.50)	(21.10, 56.31)
Week 16	29	15 (51.72)	(32.53, 70.55)
Week 24	30	14 (46.67)	(28.34, 65.67)
Week 36	19	14 (73.68)	(48.80, 90.85)
Week 52	14	8 (57.14)	(28.86, 82.34)
Last evaluation^a^	47	21 (44.68)	(30.17, 59.88)

*Note*: The Clopper–Pearson method was used for the calculation of 95% CI.

Abbreviations: CI, confidence interval; DLQI, Dermatology Life Quality Index; *N*, number of evaluable patients.

^a^ Last time point from the day after the start of secukinumab treatment to week 52. Patients with data at the start of secukinumab treatment and the last evaluation were included.

## 4 DISCUSSION

This surveillance study was conducted in clinical practice to further evaluate the safety and effectiveness of secukinumab in Japanese patients with GPP until week 52, and patients were followed up to 156 weeks from the start of secukinumab treatment.

During the 52 weeks observation period, incidences of AEs and adverse reactions were 51.58% and 35.79%, respectively, and were lower than the results reported in a previous clinical study in patients with GPP.^16^ The safety of long‐term secukinumab use was also examined in another study of plaque psoriasis, and the reported incidence of AEs was comparable with the result from this surveillance study.^21^ The trend in AE observed was similar between this surveillance and other clinical studies^16^, ^21^, ^22^, ^23^ in GPP, PPP, and plaque psoriasis. The common AE observed in other clinical studies was nasopharyngitis (the incidence was 2.11% in this surveillance).^16^, ^21^, ^22^, ^23^ The most common AE reported in this surveillance was GPP (verbatim events: worsening of the underlying disease), which was observed as the second most common AE in the clinical study of secukinumab for moderate to severe PPP.^23^ A lower incidence in SAE could also be seen; the incidence was 6.32%, lower than the results from previous clinical studies examining GPP and PPP, which ranged from 12% to 25%.^16^, ^23^ Considering the similar trend of AEs and SAEs, no new safety concerns were detected with the use of secukinumab. The incidences of AEs and adverse reactions of the priority investigation items were lower than those reported in a Japanese clinical study^16^ except for fungal infection, which was not reported in the clinical study. The outcomes of six patients with fungal infection were resolving or resolved, and no safety concern was detected. One case each of delayed development of serious infection (aspiration pneumonia) and breast cancer stage II was observed during the observation period (1006 days and 374 days, respectively, after the start of secukinumab treatment). The investigators ruled out secukinumab as the potential cause of serious infection; however, the breast cancer event was considered to be related to secukinumab. This implies no trend in the increasing incidences of specific AEs from long‐term secukinumab treatment, as also observed in other long‐term studies of plaque psoriasis,^21^, ^22^ but findings need to be interpreted with caution, as only one case each was reported. The history of biologics in patients with GPP did not affect the incidence of AEs, SAEs, adverse reactions, events that led to discontinuation, and AEs and adverse reaction in priority investigation items.

White cell count, neutrophil, and CRP were evaluated in the safety analysis population. The mean (SD) values for white cell count (/μL), neutrophil (%), and CRP (mg/dL) showed a gradual decrease starting from week 1 and remained stable from week 16 through week 52. This decrease was also reported in other studies.^24^, ^25^ These inflammatory biomarkers are associated with JDA severity criteria for pustular psoriasis, therefore the decrease in these biomarkers can also contribute to the improvement of pustular psoriasis.

The effectiveness of secukinumab was assessed and proven from global impression of change, JDA total score and severity category, PASI score and response rate, BSA, DLQI total score, and proportion of DLQI total score with 0 or 1. The increase in the response rate and improvement in the JDA total score (category) were similarly observed in a Japanese clinical study.^16^ A comparable improvement in PASI score was observed in a Japanese clinical study^16^ and phase 3 trials for plaque psoriasis,^21^, ^22^ and an improvement in DLQI score was also observed in overseas studies.^21^, ^23^

No patient characteristics were observed to be risk factors for adverse reactions or response rate of secukinumab. Considering the consistency with the result from a Japanese clinical study,^16^ patient characteristics did not seem to affect the effectiveness or safety of secukinumab in GPP.

This surveillance study was an observational study without a control group and did not collect information on patients who were not exposed to secukinumab. This was the limitation for the postulation of the causality between secukinumab exposure and the observed results. Moreover, it is important to exercise caution when interpreting the results in direct comparison with previous clinical studies^16^ because there may be variations in patient characteristics and a limited number of patients in the current surveillance. Considering these limitations, the findings from this surveillance indicate that there are no new safety concerns or issues that require attention regarding the long‐term use of secukinumab in patients with GPP, therefore, currently, there is no requirement to consider any additional measures. Moreover, secukinumab showed sustained effectiveness through 52 weeks of treatment in patients with GPP in Japan.

## CONFLICT OF INTEREST STATEMENT

A.F. and N.S. are employees of Novartis Pharma K.K. There are no financial or personal relationships between authors and others that could bias the work set out in the manuscript as declared in the statement.

## ETHICS STATEMENT

The surveillance followed the ethical policies of this journal and the institution's ethics committees.

## Supporting information


**Data S1.** Supporting information.
