# Supplementary material for: Safety and effectiveness of secukinumab in Japanese patients with generalized pustular psoriasis: A post‐marketing surveillance
Source: J Dermatol. 2025 Apr 3;52(5):773–86. doi: 10.1111/1346-8138.17648 (PMC12056282; doi:10.1111/1346-8138.17648)
Supplement: Supplementary file 1 — Data S1. Supporting information. [file JDE-52-773-s001.docx]

**Supplementary Tables**

## Table S1. Patient discontinuation (safety analysis population)

| **Breakdown** | **Number of patients (%)**  **N = 95** |
| --- | --- |
| **Patients who completed the 52-week observation** | 86 (90.53) |
| **Patients who did not complete the 52-week observation** | 9 (9.47) |
| **Reason for discontinuation** | |
| Withdrawal of consent by the patient | 3 (3.16) |
| Failure to return after the first visit (including hospital transfer) | 0 (0.00) |
| Failure to return before completion (including hospital transfer) | 5 (5.26) |
| Deaths | 1 (1.05) |
| Others | 0 (0.00) |
| Unknown | 0 (0.00) |
| N, total number of patients.  Patients with multiple reasons for discontinuation were counted in the respective reasons. | |

## Table S2. Observation durations and secukinumab administration (safety analysis population)

| **Variables** | **Category** | **n (%), N = 95** |
| --- | --- | --- |
| **Observation duration^†^, n (%)** | Entire period | 95 (100.00) |
|  | >4 weeks | 95 (100.00) |
|  | >16 weeks | 93 (97.89) |
|  | >24 weeks | 90 (94.74) |
|  | >32 weeks | 88 (92.63) |
|  | >40 weeks | 87 (91.58) |
|  | >48 weeks | 87 (91.58) |
|  | >52 weeks | 87 (91.58) |
| **Observation duration (day), n = 95** | Mean (SD) | 346.2 (64.87) |
|  | Median (range) | 365.0 (42‑365) |
|  | Total observation duration (patient-year) | 90.0 |
| **Secukinumab treatment duration^‡^,  n (%)** | Entire period | 95 (100.00) |
|  | >4 weeks | 87 (91.58) |
|  | >16 weeks | 80 (84.21) |
|  | >24 weeks | 73 (76.84) |
|  | >32 weeks | 68 (71.58) |
|  | >40 weeks | 66 (69.47) |
|  | >48 weeks | 58 (61.05) |
|  | >52 weeks | 18 (18.95) |
|  | Unknown/not recorded | 2 (2.11) |
| **Secukinumab treatment duration (days), n = 93** | Mean (SD) | 278.1 (120.41) |
|  | Median (range) | 344.0 (8‑365) |
|  | Total exposure duration (patient-year) | 70.8 |
| **Starting dose of secukinumab**  **(per administration), n (%)** | 150 mg | 5 (5.26) |
|  | 300 mg | 90 (94.74) |
| **Most frequent dose of secukinumab**  **(per administration), n (%)** | 150 mg | 3 (3.16) |
|  | 300 mg | 92 (96.84) |
| **Total number of secukinumab administration, n = 95** | Mean (SD) | 13.1 (4.32) |
|  | Median (range) | 15.0 (2‑17) |
| **Secukinumab dose**  **increase/reduction, n (%)** | No dose increase/reduction | 87 (91.58) |
|  | Dose increase only | 3 (3.16) |
|  | Dose reduction only | 4 (4.21) |
|  | Dose increase and reduction | 1 (1.05) |
| **Self-administration^§^, n (%)** | No | 51 (53.68) |
|  | Yes | 44 (46.32) |
| N, total number of patients; n, number of patients; SD, standard deviation.  ^†^Observation duration = last observation day − first treatment day + 1.  ^‡^Secukinumab treatment duration = last administration day − first administration day + 1.  ^§^Patients who had self-administration at least once during the secukinumab administration period were classified to “Yes.” | | |

## Table S3. Reasons for switching by type of biologics administered before start of secukinumab (safety analysis population)

| **Drug** | **Number of patients treated previously**  **N = 95** | **Reason for** **switching, n (****%)^†^** | | | | |
| --- | --- | --- | --- | --- | --- | --- |
|  |  | **Lack of**  **efficacy** | **AE** | **Patient’s**  **will** | **Financial reasons** | **Others** |
| Infliximab | 35 | 18 (51.43) | 14 (40.00) | 1 (2.86) | 0 (0.00) | 1 (2.86) |
| Anti‑IL‑17A antibody | 0 | - (-) | - (-) | - (-) | - (-) | - (-) |
| Anti-IL-17 receptor  antibody | 0 | - (-) | - (-) | - (-) | - (-) | - (-) |
| Others | 15 | 10 (66.67) | 1 (6.67) | 0 (0.00) | 0 (0.00) | 2 (13.33) |
| AE, adverse event; IL, interleukin; N, total number of patients; n, number of evaluable patients.  Patients previously treated with multiple biologics were counted in the respective biologics.  ^†^Numbers of patients previously treated with the respective biologics were used as denominators to calculate the proportions. | | | | | | |

## Table S4. Number of days from the last date of prior biologics to the start of secukinumab (safety analysis population)

| **Drug names** | **Number of days to switch to secukinumab^†^** | | |
| --- | --- | --- | --- |
|  | **Number of patients**  **N = 95** | **Mean (****SD)** | **Median (range)** |
| Infliximab | 24 | 350.6 (639.10) | 57.0 (28‑2226) |
| Anti-IL-17A antibody | 0 | - | - |
| Anti-IL-17 receptor antibody | 0 | - | - |
| Others | 8 | 185.5 (396.48) | 43.5 (15‑1164) |
| IL, interleukin; N, total number of patients; SD, standard deviation.  Drugs used for “pustular psoriasis” were considered.  Patients previously treated with multiple biologics were counted in the drug that was most recently used.  ^†^Number of days from the last dates of biologics administered before the start of secukinumab treatment to the start of secukinumab. | | | |

## Table S5. Concomitant drugs and therapies used during the observation period and the secukinumab treatment period (safety analysis population)

| **Concomitant drugs/therapies used** | **During observation period, n (%)**  **N = 95** | **During secukinumab treatment period, n (%)**  **N = 95** |
| --- | --- | --- |
| **Biologics** | 9 (9.47) | 1 (1.05) |
| Infliximab | 1 (1.05) | 0 (0.00) |
| Anti-IL-17A antibody | 2 (2.11) | 0 (0.00) |
| Anti-IL-17 receptor antibody | 5 (5.26) | 0 (0.00) |
| Others | 2 (2.11) | 1 (1.05) |
| **Non-biologics** | 80 (84.21) | 78 (82.11) |
| Cyclosporine | 13 (13.68) | 11 (11.58) |
| Etretinate | 21 (22.11) | 20 (21.05) |
| Topical steroid | 58 (61.05) | 54 (56.84) |
| Others | 54 (56.84) | 53 (55.79) |
| **Other drugs^†^** | 72 (75.79) | 71 (74.74) |
| **Phototherapy^‡^** | 2 (2.11) | NA |
| Oral PUVA | 0 (0.00) | NA |
| External PUVA | 0 (0.00) | NA |
| PUVA bath | 0 (0.00) | NA |
| UVB | 0 (0.00) | NA |
| Narrow-band UVB | 1 (1.05) | NA |
| Others | 1 (1.05) | NA |
| **GCAP^§^** | 5 (5.26) | NA |
| **Concomitant therapies**  **(not for pustular psoriasis)^¶^** | 4 (4.21) | 2 (2.11) |
| GCAP, granulocyte and monocyte adsorption apheresis; IL, interleukin; N, total number of patients; NA, not applicable; n, number of patients; PUVA, psoralen plus ultraviolet A; UVB, ultraviolet B.  ^†^Drugs for purposes other than pustular psoriasis treatment.  ^‡^Phototherapy for pustular psoriasis.  ^§^GCAP for pustular psoriasis.  ^¶^Concomitant therapies for purposes other than pustular psoriasis treatment (other than medication,  phototherapy [PUVA, UVB], GCAP) | | |

## Table S6. Incidence of SAEs (by SOC, PT; safety analysis population)

| **SOC**  **PT** | **Number of patients with SAE (%)**  **N = 95** |
| --- | --- |
| **Total** | 12 (12.63) |
| **Infections and infestations** | 4 (4.21) |
| Oral candidiasis | 1 (1.05) |
| Pneumonia staphylococcal | 1 (1.05) |
| Sepsis | 1 (1.05) |
| Septic shock | 1 (1.05) |
| Tonsilitis | 1 (1.05) |
| **Neoplasms benign, malignant, and unspecified (including cysts and polyps)** | 3 (3.16) |
| Breast cancer stage II | 1 (1.05) |
| Pancreatic carcinoma recurrent | 1 (1.05) |
| Hepatocellular carcinoma* | 1 (1.05) |
| **Endocrine disorders** | 1 (1.05) |
| Adrenal insufficiency | 1 (1.05) |
| **Gastrointestinal disorders** | 2 (2.11) |
| Colitis ulcerative | 1 (1.05) |
| Intestinal obstruction | 1 (1.05) |
| **Skin and subcutaneous tissue disorders** | 1 (1.05) |
| Erythema | 1 (1.05) |
| Pruritus | 1 (1.05) |
| **General disorders and administration site conditions** | 1 (1.05) |
| Pyrexia | 1 (1.05) |
| N, total number of patients; PT, preferred term; SAE, serious adverse event; SOC, system organ class.  A patient with multiple events (PT) was counted only once.  SOC are shown in the order of international consensus. PT is shown in the descending order of incidences, followed by order of PT codes.  *The patient was diagnosed with hepatocellular carcinoma 149 days after the start of secukinumab treatment (9 days after the last dose) and the treatment was discontinued. The patient died 1234 days after its onset. The investigators ruled out any causal relationship with secukinumab.  MedDRA/J version 24.1 | |

**Table S7**. Incidence of adverse reactions (by SOC, PT; safety analysis population)

| **SOC**  **PT** | **Number of patients with**  **adverse reaction (%)**  **N = 95** |
| --- | --- |
| **Total** | 34 (35.79) |
| **Infections and infestations** | 9 (9.47) |
| Oral candidiasis | 3 (3.16) |
| Folliculitis | 2 (2.11) |
| Nasopharyngitis | 1 (1.05) |
| Pneumonia staphylococcal | 1 (1.05) |
| Sepsis | 1 (1.05) |
| Septic shock | 1 (1.05) |
| Skin candida | 1 (1.05) |
| Tinea infection | 1 (1.05) |
| Candida infection | 1 (1.05) |
| **Neoplasms benign, malignant, and unspecified  (including cysts and polyps)** | 1 (1.05) |
| Breast cancer stage II | 1 (1.05) |
| **Blood and lymphatic system disorders** | 1 (1.05) |
| Elliptocytosis | 1 (1.05) |
| **Psychiatric disorders** | 1 (1.05) |
| Insomnia | 1 (1.05) |
| **Nervous system disorders** | 1 (1.05) |
| Headache | 1 (1.05) |
| Hypoesthesia | 1 (1.05) |
| **Vascular disorders** | 2 (2.11) |
| Hypertension | 2 (2.11) |
| **Respiratory, thoracic, and mediastinal disorders** | 2 (2.11) |
| Interstitial lung disease | 1 (1.05) |
| Upper respiratory tract infection | 1 (1.05) |
| **Gastrointestinal disorders** | 1 (1.05) |
| Colitis ulcerative | 1 (1.05) |
| **Hepatobiliary disorders** | 1 (1.05) |
| Liver disorder | 1 (1.05) |
| **Skin and subcutaneous tissue disorders** | 13 (13.68) |
| Pustular psoriasis | 4 (4.21) |
| Rash | 3 (3.16) |
| Acne | 1 (1.05) |
| Drug eruption | 1 (1.05) |
| Erythema | 1 (1.05) |
| Pain of skin | 1 (1.05) |
| Pruritus | 1 (1.05) |
| Seborrheic dermatitis | 1 (1.05) |
| Urticaria | 1 (1.05) |
| **General disorders and administration site conditions** | 3 (3.16) |
| Drug ineffective | 1 (1.05) |
| Malaise | 1 (1.05) |
| Therapeutic response decreased | 1 (1.05) |
| **Investigations** | 7 (7.37) |
| Neutrophil count decreased | 2 (2.11) |
| White blood cell count decreased | 2 (2.11) |
| Blood triglycerides increased | 1 (1.05) |
| N, total number of patients; PT, preferred term; SOC, system organ class.  A patient with multiple events (PT) was counted only once.  SOC are shown in the order of international consensus. PT is shown in the descending order of incidences, followed by the order of PT codes.  MedDRA/J version 24.1 | |

## Table S8. Data on the performance of hepatitis tests and tuberculosis tests (safety analysis population)

| **Test** | **Performed at least once during observation period** |
| --- | --- |
|  | **n (****%)^†^, N = 95** |
| Hepatitis test | 44 (46.32) |
| HBs antigen | 30 (31.58) |
| HBs antibody | 31 (32.63) |
| HBc antibody | 23 (24.21) |
| HBe antigen | 5 (5.26) |
| HBe antibody | 5 (5.26) |
| HBV-DNA | 20 (21.05) |
| Tuberculosis test | 68 (71.58) |
| Tuberculin reaction test | 2 (2.11) |
| QuantiFERON test | 6 (6.32) |
| T-Spot | 32 (33.68) |
| Chest X-ray | 60 (63.16) |
| Chest CT | 20 (21.05) |
| CT, computed tomography; HBc, hepatitis B core; HBe, hepatitis B e; HBs, hepatitis B surface; HBV, hepatitis B virus; N, total number of patients; n, number of patients.  †Patients with a negative or positive result from the performed tests were counted. | |

**Supplementary Figure**

## Figure S1. Changes in the CRP level (box plot; safety analysis population)


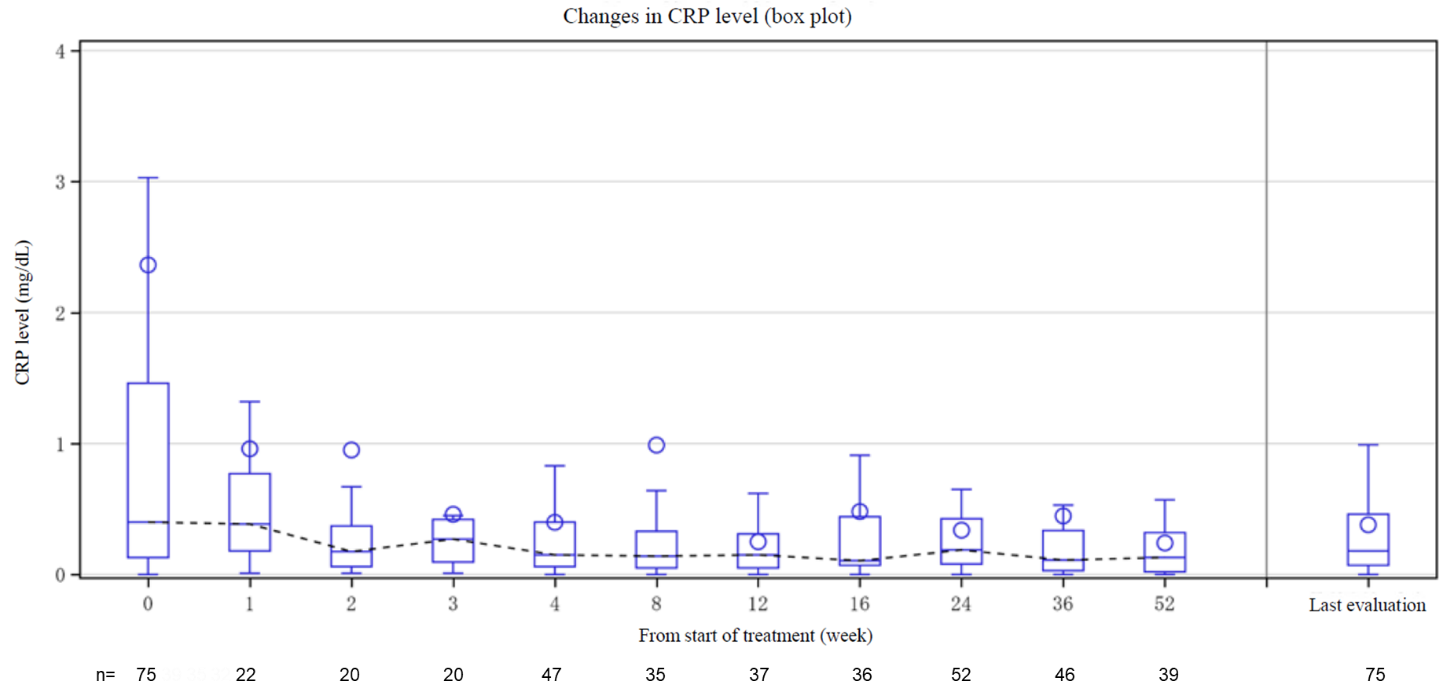
Օ, Mean CRP value; CRP, C-reactive protein.
Patients with results at the start of treatment and the last evaluation were included.
